# Reviewing a Decade of Outpatient Tropical Medicine in Houston, Texas

**DOI:** 10.4269/ajtmh.21-1059

**Published:** 2022-02-28

**Authors:** Julika Kaplan, Fernando Hernandez Centeno, Jesica Hayon, Maria Elena Bottazzi, Peter J. Hotez, Jill E. Weatherhead, Eva Clark, Laila Woc-Colburn

**Affiliations:** ^1^ Department of Internal Medicine, Baylor College of Medicine, Houston, Texas;; ^2^ Department of Pediatrics, Baylor College of Medicine, Houston, Texas;; ^3^ School of Medicine, Baylor College of Medicine, Houston, Texas;; ^4^ Department of Medicine, Section of Infectious Diseases, Baylor College of Medicine, Houston, Texas;; ^5^ National School of Tropical Medicine, Baylor College of Medicine, Houston, Texas;; ^6^ Department of Molecular Virology and Microbiology, Baylor College of Medicine, Houston, Texas;; ^7^ Health Services Research, Michael E. DeBakey VA Health Services Research Center of Innovations, Houston, Texas;; ^8^ Department of Medicine, Division of Infectious Diseases, Emory University School of Medicine, Atlanta, Georgia

## Abstract

Tropical diseases cause significant morbidity among the world’s poorest populations. Although more common in low- and middle-income countries, tropical diseases are also found among underserved populations living in high-income countries such as the United States. The National School of Tropical Medicine at Baylor College of Medicine and the Harris Health System founded a tropical medicine clinic—the Harris Health Tropical Medicine Clinic (HHTMC)—in Houston in 2011 in response to tropical disease-related morbidity in Texas. We conducted a retrospective chart review of a sample of patients older than 18 years of age who were referred to the HHTMC between October 2011 and January 2020. Of the 523 patients reviewed, 185 (35.4%) had mycobacterial infections, 184 (35.2%) had parasitic infections, 38 (7.3%) had fungal infections, 16 (3.1%) had eosinophilia without a confirmed clinical diagnosis, 28 (5.4%) had bacterial infections, and 13 (2.5%) had viral infections. The most common infections overall were extrapulmonary and latent tuberculosis (*n* = 169), neurocysticercosis (*n* = 78), strongyloidiasis (*n* = 28), Chagas disease (*n* = 25), and schistosomiasis (*n* = 12). The epidemiology of tropical diseases in the United States is understudied at national and regional levels. This 10-year retrospective study contributes to bridging this knowledge gap by detailing the frequencies of tropical disease diagnoses made at the HHTMC in Houston, TX. These data highlight areas for advancement in the field of tropical medicine within the United States, such as improving front-line health-care provider education; establishing tropical medicine clinics in areas of high prevalence such as the Gulf Coast, Appalachia, and urban areas; and developing comprehensive, systematic national tropical disease screening programs and patient registries.

## INTRODUCTION

Tropical infectious diseases cause significant morbidity among the world’s poorest populations. These infections include “the big three”—HIV, malaria, and tuberculosis—as well as the “neglected” tropical diseases (NTDs). ^1^ Chronic tropical infections often lead to growth and cognitive impairment in children, and chronic illnesses in adulthood. NTDs alone cause more than 60 million disability-adjusted life-years annually in low- and middle-income countries (LMICs). ^2^ Although tropical diseases are typically associated with LMICs, they are also found among underserved populations living in high-income countries such as the United States. ^3^ The concept of recognizing the tropical disease burden in relatively wealthy nations is described via the “blue marble health” framework. ^4^ In the United States, regional tropical disease prevalence is driven by several major factors, including 1) human migration and urbanization, 2) socioeconomic status (which is related directly to access to health care), 3) climate change, and 4) the presence of suitable vectors. Many of these factors are present in the southern United States and Texas, and contribute to the frequency of tropical disease diagnoses in this region, ^5^ such as autochthonous arbovirus infections, Chagas disease, ^6^ neurocysticercosis (NCC), ^7^ intestinal parasite infections, ^8^ tuberculosis, ^9^ and murine typhus.

In 2011, in response to the growing recognition of morbidity related to NTDs in southeastern Texas, the National School of Tropical Medicine (NSTM) at Baylor College of Medicine founded an outpatient referral tropical medicine clinic in partnership with the Harris Health System to provide outpatient care for patients with tropical diseases. ^10^ Since its establishment, the Harris Health Tropical Medicine Clinic (HHTMC) has provided specialized care for hundreds of patients with tropical diseases in the greater Houston area, including recent immigrants, long-term community members, and travelers.

The infections most seen at the HHTMC—extrapulmonary and latent tuberculosis, NCC, strongyloidiasis, Chagas disease, and schistosomiasis—are likely underrecognized and underdiagnosed in the greater Houston area. For example, *Strongyloides stercoralis* is endemic in many parts of the United States, including the Gulf Coast and Appalachia ^11^; but, despite the likely large burden of endemic disease in the United States, ^12^ no comprehensive national or even recent large regional seroprevalence studies or screening guidelines exist. Similarly, we suspect that case detection of Chagas disease is poor as a result of inadequate provider knowledge of screening indications, the prolonged asymptomatic nature of the disease, and poor sensitivity of available screening tests. This study contributes to the existing epidemiological data regarding tropical disease prevalence in the Houston area and highlights the need for clinics such as the HHTMC.

## METHODS

### Description of study site.

The HHTMC exists within the Harris Health System, which is a health-care safety net that serves approximately 1 million Harris County residents who are uninsured or underinsured (> 50% of patients are uninsured). ^13^ The Harris Health patient population is approximately 58% Hispanic, 24% Black/African American, 10% White/Caucasian, and 8% Asian/Other. ^13^ Houston—the largest city in Harris County and in Texas—is home to more than 1 million immigrants, of whom more than 20% live below the federal poverty level. Most immigrants to Houston originated in Latin America, although a large and growing number come from African and Asian countries. ^14^ The HHTMC captures not only NTDs, but also travel and migration-related diseases, as well as diseases with high burdens in tropical and subtropical areas. ^15^

The HHTMC is open one-half day each week and is staffed by three infectious diseases physicians who specialize in tropical medicine. A patient must have a referral to be seen at the HHTMC, and the referral must describe a suspected diagnosis of a tropical illness and include supporting laboratory tests, imaging, or description of physical examination findings. All referrals are screened by a tropical medicine provider to evaluate their appropriateness for the clinic. Many patients already have a tropical disease diagnosis at the time of referral (thus are referred to the HHTMC for outpatient follow-up and management), but some diagnoses are made or confirmed by HHTMC providers. Patients not typically seen at the HHTMC include those requiring pre-travel assessment, those with pulmonary tuberculosis, and those with HIV who already have an HIV provider within the Harris Health System. Patients with pulmonary tuberculosis are seen primarily at the Harris Health System’s Pulmonary Clinic; however, patients with extrapulmonary tuberculosis are seen at the HHTMC.

### Study design and data collection.

Harris Health System’s Information Technology Service extracted the electronic medical records of all patients seen by the three tropical medicine providers who staffed the HHTMC between 2011 and 2020; we did not use International Classification of Diseases codes for patient selection. We included patients 18 years or older seen at the HHTMC between October 2011 and January 2020. Four authors (J. K., F. C., J. H., and E. C.) reviewed patients’ electronic medical records systematically, extracted data (including demographic information, reasons for referral, final diagnoses, and exposure histories), and entered the data systematically into an electronic REDCap database (Nashville, TN). These four authors discussed and resolved collectively any chart review discrepancies. Some patients had more than one diagnosis, thus the total number of diagnoses described is greater than the total number of patients.

We classified patients into the following three categories based on their health insurance status: 1) “Gold Card,” meaning the Harris Health Financial Assistance Program (formerly known as the “Gold Card”); 2) “private,” meaning employment-based health insurance, federal marketplace-based health insurance, social insurance programs such as Medicare, and social welfare programs such as Medicaid; or 3) “self-pay,” meaning no health insurance. The Gold Card is a unique financial assistance program with flexible eligibility criteria available to low-income Harris County residents, including those who are homeless, living with friends or family in the county, or undocumented immigrants. Benefits are tiered based on income level. ^16^

We categorized countries of origin into six regions based on the United Nations M49 standard for world regions. ^17^ For simplicity, within this classification system, we refer to the “Latin America and the Caribbean region” as “Latin America.” Eosinophilia was defined as an absolute value greater than 500 cells/μL.

### Data analysis.

We described categorical variables using frequencies and percentages, and continuous variables using medians and ranges. We analyzed the data using GraphPad Prism 9 software (San Diego, CA).

### Ethics statement.

This protocol (H-42248) was reviewed and approved by Baylor College of Medicine’s institutional review board.

## RESULTS

### Patient demographics.

Of the 523 patients included in the study, the majority were women (*n* = 292, 55.8%), were originally from Latin America (*n* = 288, 55.1%), and had lived in the United States for 10 years or more (*n* = 286, 54.7%) (Table 1). Most patients lived in Harris County (*n* = 492, 94.1%) and had health-care funding either from the Gold Card (*n* = 255, 48.8%) or private insurance (*n* = 198, 37.9%).

**Table 1:** Patient demographics (*N* = 523)

Demographic	Value
Age, y; median (range)	47 (19–89)
Female gender, *n *(%)	292 (55.8)
Region of origin,* *n* (%)	
Africa	50 (9.6)
Asia	67 (12.8)
Europe	3 (0.6)
Latin America	288 (55.1)
North America	97 (18.5)
Unknown	18 (3.4)
Race/ethnicity, *n* (%)	
Hispanic	310 (59.3)
Black	83 (15.9)
White	49 (9.4)
Middle Eastern	27 (5.2)
Asian/Pacific Islander	35 (6.7)
Other/unknown	19 (3.6)
Time living in the United States, *n* (%)	
< 1 year	26 (5.0)
1 to < 5 years	39 (7.5)
5 to < 10 years	49 (9.4)
10 years or more	286 (54.7)
Unknown	123 (23.5)
Healthcare coverage,† *n* (%)	
Gold Card	255 (48.8)
Private	198 (37.9)
Self-pay	92 (17.6)
Other	32 (6.1)
County of residence, *n *(%)	
Harris, TX	492 (94.1)
Other, in Texas	24 (4.6)
Other, outside of Texas	6 (1.1)
Outside the United States	1 (0.2)

* Regions were defined with the United Nations geoscheme, with sub-regional divisions used for the Americas. ^17^

† The sum exceeds the total number of patients because some patients had more than one type of health-care coverage.

### Overview of tropical medicine diagnoses.

**Figure 1.**
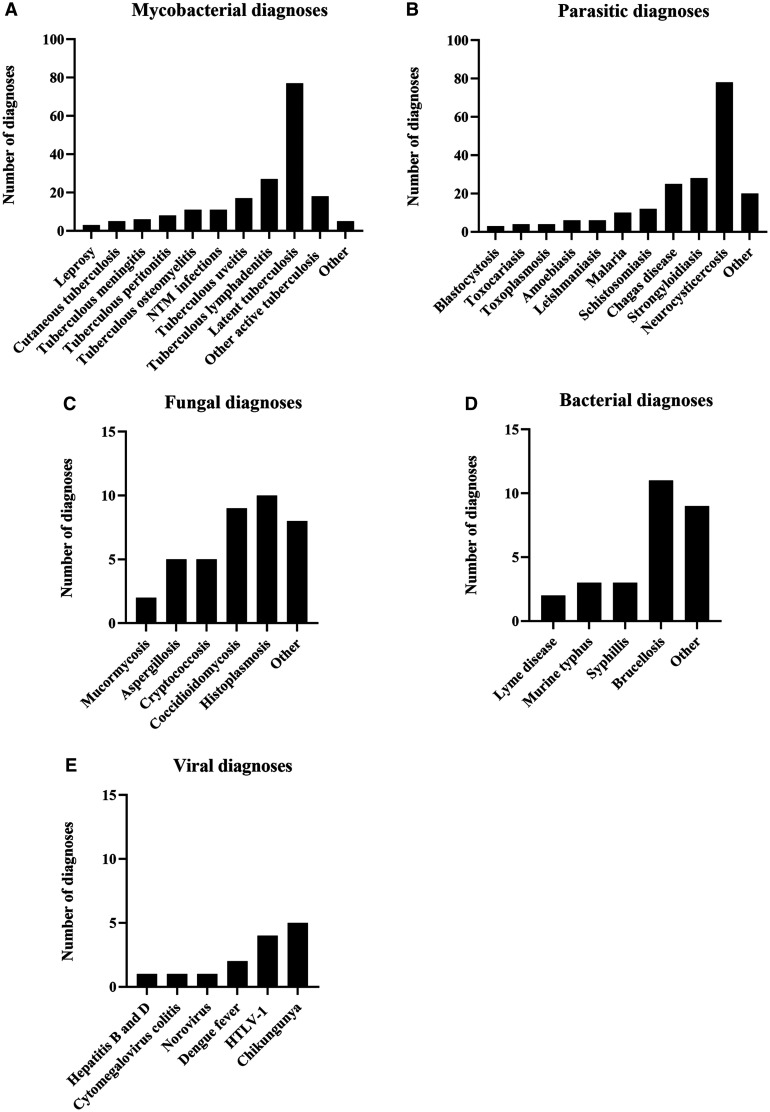
Final infection diagnoses by disease category. “Other active tuberculosis” includes tuberculous mastitis, tuberculous tenosynovitis, tuberculous hepatitis, tuberculous aortitis, and Poncet’s disease.

We identified 448 different pathogen diagnoses among the 523 patients. Of these, 185 patients (35.4%) had mycobacterial infections, 180 (34.4%) had parasitic infections, 38 (7.3%) had fungal infections, 28 (5.4%) had bacterial infections, and 13 (2.5%) had viral infections (Figure 1). In addition, 16 patients (3.1%) had eosinophilia without a clear etiology. There were 83 patients (15.7%) with other diagnoses who did not fit into these categories, and 12 patients (2.3%) with unknown diagnoses. The most common infections seen were extrapulmonary and latent tuberculosis (*n* = 169), NCC (*n* = 78), strongyloidiasis (*n* = 28), Chagas disease (*n* = 25), and schistosomiasis (*n* = 12) (Supplemental Table 1).

Fourteen of the patients included in this study had nontuberculous mycobacterial (NTM) infections: six with *Mycobacterium avium* complex, four with *M. fortuitum*, three with *M. leprae*, and one with *M. chelonae*. Patients with NTM infections were diagnosed in a variety of ways. Some patients were referred to the HHTMC because of suspected NTM diagnosis only, whereas others already had confirmed NTM specimens and were referred for management.

Three of the patients included in this study had HIV/AIDS. One of these patients had latent tuberculosis, one had *Entamoeba histolytica* infection, and one had both toxoplasmosis and syphilis. Among the patients with extrapulmonary tuberculosis, none were HIV positive.

The number of patients seen per year remained relatively constant over time (Supplemental Figure 1).

### Description of diagnoses by disease category.

#### Mycobacterial diagnoses.

**Figure 2.**
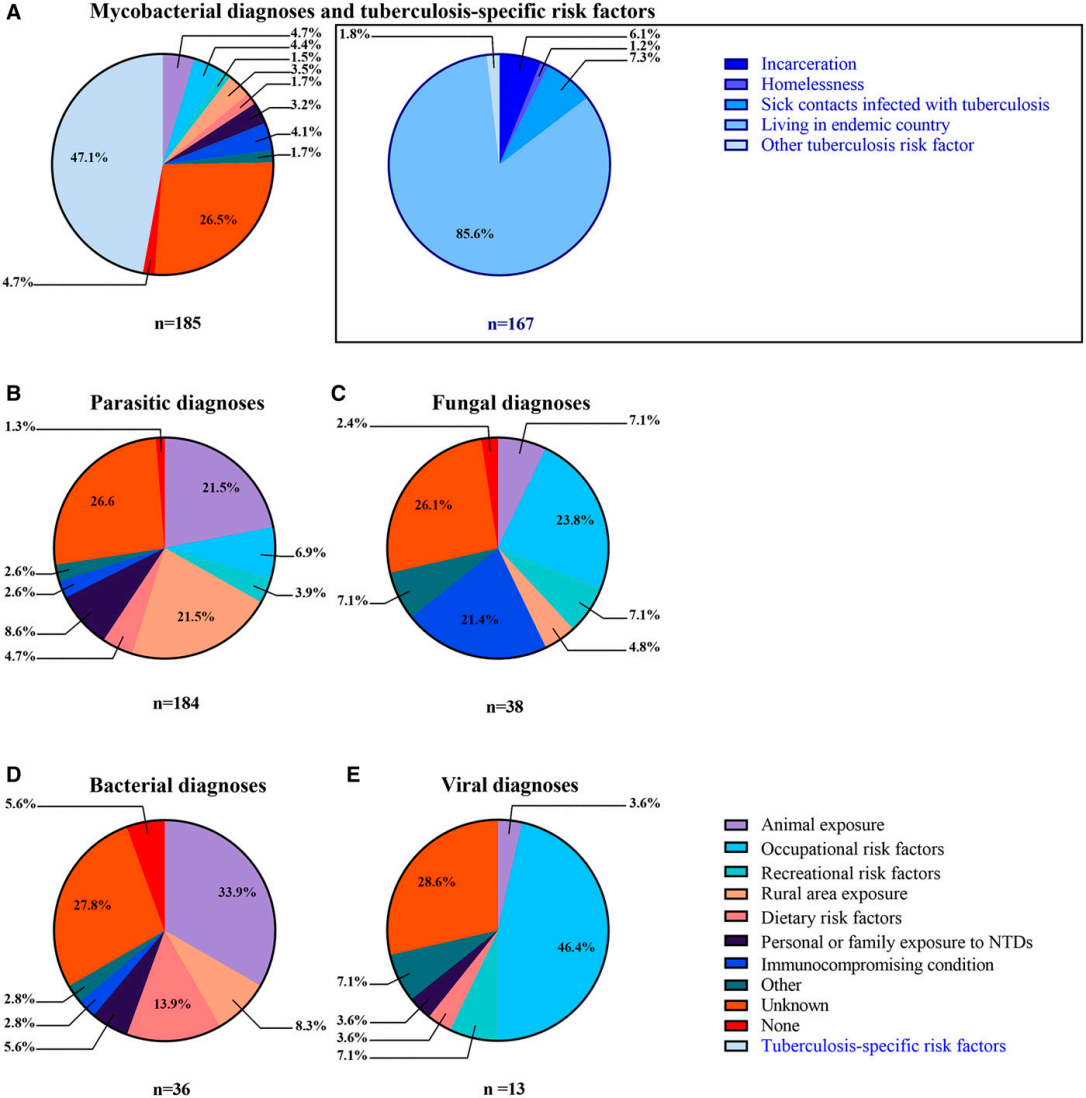
Proportion of risk factors for each diagnostic category. The inset represents tuberculosis-specific risk factors and includes only patients with Mycobacterium tuberculosis infections. “Occupational risk factors” include medical, agricultural, veterinary, archaeological, military, and refugee work. “Recreational risk factors” include fishing, hunting, swimming, and gardening. “Dietary risk factors” include consumption of unpasteurized dairy and uncooked meat. “Immunocompromising conditions” include HIV, and oncological and rheumatological conditions. NTDs = neglected tropical diseases. This figure appears in color at www.ajtmh.org.

Most of the 185 mycobacterial diagnoses were extrapulmonary *Mycobacterium tuberculosis* (*n* = 83, 44.9%). Seventy-seven patients had latent tuberculosis and 14 had nontuberculous mycobacterial infections. Of the 165 patients with either extrapulmonary or latent *M. tuberculosis* infections, the most common risk factor was residence in a tuberculosis-endemic country for 10 years or longer (Figure 2A). The majority of risk factors reported by patients with mycobacterial infections included exposure to animals and occupational risk factors (Figure 2A). Most patients diagnosed with mycobacterial infections were born in Latin America (*n* = 96, 51.9%), but others were from Asia (*n* = 35, 18.9%), North America (*n* = 25, 13.5%), or Africa (*n* = 22, 11.9%). Only one patient was from Europe. Thirty-two patients (17.2%) reported traveling to the following continents: Latin America (*n *= 24, 13.0%), North America (*n *= 17, 9.2%), Asia (*n *= 10, 5.4%), Africa (*n *= 8, 4.3%), and Europe (*n *= 6, 3.2%). Of these patients, 15 traveled outside the United States within the 2 years prior to their initial clinic visits.

#### Parasitic diagnoses.

Of the 184 patients with parasitic infections, NCC was the most common diagnosis (*n *= 78, 42.4%), followed by strongyloidiasis (*n *= 28, 15.2%) and Chagas disease (*n *= 25, 13.6%). Other parasitic diagnoses included schistosomiasis (*n *= 12, 6.5%), malaria (*n *= 10, 5.4%), and leishmaniasis (*n *= 6, 3.3%). Many of the patients diagnosed with parasitic infections reported animal exposure (*n *= 53, 28.8%) and residence in rural areas (*n *= 52, 28.3%) (Figure 2B). Less commonly, patients with parasitic infections reported occupational (*n *= 18, 9.8%), dietary (*n *= 11, 6.0%), and recreational (*n *= 9, 4.9%) exposures. Most of the patients with parasitic infections were born in Latin America (*n *= 137, 74.5%). Fewer originated from Africa (*n *= 22, 12.0%), North America (*n *= 12, 6.5%), and Asia (*n *= 9, 4.9%). Sixty-one patients (33.2%) reported traveling to Latin America (*n *= 37, 20.1%), Africa (*n *= 13, 7.2%), North America (*n *= 10, 5.4%), Asia (*n *= 6, 3.3%), and Europe (*n *= 3, 1.7%). Of these patients, 25 traveled outside the United States within the 2 years prior to their initial clinic visits.

#### Fungal diagnoses.

Thirty-eight patients had fungal infections, including histoplasmosis (*n *= 10, 26.3%), coccidioidomycosis (*n *= 9, 23.7%), cryptococcosis (*n *= 5, 13.2%), aspergillosis (*n *= 5, 13.2%), and mucormycosis (*n *= 2, 5.3%). Patients with fungal infections most commonly reported occupational (*n *= 10, 26.3%) risk factors (Figure 2C). Nine patients had immunocompromising health conditions including diabetes, cancer, or autoimmune conditions (Figure 2C). Most patients were born in Latin America (*n *= 20, 52.6%), but some were from North America (*n *= 11, 28.9%) and Africa (*n *= 3, 7.9%); only one was from Asia (2.6%). Six patients (15.8%) reported travel, mostly within North America (*n *= 5, 13.2%) and within the past 6 months (*n *= 4, 10.5%).

#### Bacterial diagnoses.

Twenty-eight patients had bacterial infections including brucellosis (*n *= 11, 39.3%), murine typhus (*n *= 3, 10.7%), syphilis (*n *= 3, 10.7%), Lyme disease (*n *= 2, 7.1%), Whipple disease (*n *= 1, 3.6%), and rheumatic fever (*n *= 1, 3.6%). The most common risk factor among patients with bacterial infections was animal exposure (*n *= 12, 42.9%) (Figure 2D). Most patients were born in Latin America (*n *= 19, 67.8%), and 11 patients (39.3%) reported travel, mostly to Latin America (*n *= 9, 32.1%).

#### Viral diagnoses.

Thirteen patients had viral infections, including chikungunya (*n *= 5, 38.5%), human T-lymphotropic virus (*n *= 4, 30.8%), and dengue (*n *= 2, 15.4%). Of these, two (15.4%) had recreational exposures and one (7.7%) had an animal exposure (Figure 2E). Patients with viral infections were born in Latin American (*n *= 4, 30.8%), North America (*n *= 4, 30.8%), Asia (*n *= 2, 15.4%), and Europe (*n *= 1, 7.7%). Six patients (46.2%) reported traveling to the following continents, all within the 2 years prior to clinic presentation: Latin America (*n *= 5, 38.5%), Asia (*n* = 2, 15.4%), Africa (*n *= 1, 7.7%), and Europe (*n *= 1, 7.7%). Of the five patients diagnosed with chikungunya, four had recently traveled to Latin America, and of the two patients diagnosed with dengue, one had recently traveled to Africa (Tanzania, Kenya, and South Africa) and the other had recently traveled to Mexico. Unlike the other disease categories, patients with viral diagnoses were funded most commonly through private health insurance (*n *= 7, 34.3%).

#### Eosinophilia.

Sixteen patients had diagnoses of peripheral eosinophilia (*n *= 16) of initially unclear etiology. These patients had country- and exposure-based serological testing with or without ova and parasite testing, and most never received a more specific diagnosis. One was diagnosed with giardiasis. Four received empiric treatment with ivermectin, and three of these had resolution of eosinophilia after treatment; the fourth did not return to the HHTMC after treatment. Many reported exposures to rural areas (*n *= 8, 50.0%). Seven of 16 patients with eosinophilia were born in Latin America (43.8%). Twelve patients (75%) reported travel to the following continents: Latin America (*n *= 6, 37.8%), Asia (*n *= 3, 18.8%), Africa (*n *= 2, 12.5%), and Europe (*n *= 2, 12.5%). Five had traveled within the past 5 years.

## DISCUSSION

Baylor College of Medicine’s NSTM is a unique educational entity established in 2011 to facilitate training and research in diseases of poverty in the United States. The NSTM is committed to health equity and addressing the realities of climate change, urbanization, and human migration linked to the blue marble health framework. Here we describe the role of the HHTMC within the local medical community and review the spectrum of diagnoses made at the HHTMC. The volume and diversity of the illnesses encountered reaffirm the NSTM’s founding principles.

In contrast to public health departments, refugee clinics, and travel medicine clinics, the HHTMC, as an outpatient referral clinic within a county health-care system, focuses specifically on care for patients with sub-acute and chronic tropical illnesses (Figure 1). The HHTMC primarily serves people who have lived in the Houston area for many years (Table 1) and likely acquired their tropical diseases either in their country of origin or via autochthonous local transmission. Most of the patients included in our analysis are of Latin American descent and have health-care coverage through the county safety net system (Gold Card, Table 1), which reflects the wider Harris Health patient population. ^13^ Importantly, there are certain patient populations that do not receive care at the HHTMC as a result of the structure of the Harris Health System. For example, patients with pulmonary tuberculosis are treated at Harris Health’s Pulmonology Clinic; only patients with extrapulmonary tuberculosis are seen at the HHTMC. In addition, most people with HIV, even those co-infected with tropical diseases, receive longitudinal care and treatment of tropical diseases at Harris Health HIV specialty clinics. Last, pediatric patients with tropical diseases are referred to the Texas Children’s Hospital Tropical Medicine Clinic, which was recently launched in August 2020 in partnership with and supported by the NSTM.

A broad spectrum of tropical diseases are diagnosed and managed at the HHTMC. Here we describe the most common tropical infections diagnosed at the HHTMC in the context of their regional epidemiology.

### Extrapulmonary and latent tuberculosis.

The prevalence of tuberculosis is relatively low in the United States compared with many LMICs. An estimated 13.6 million people (4.8%) living in the United States have latent tuberculosis infections, which are diagnosed more commonly among those born outside the United States. ^18^ Consequently, the U.S. Preventative Services Task Force recommends that high-risk asymptomatic adults, including those born in endemic countries, be tested for latent tuberculosis. ^19^ Although we describe 77 cases of latent tuberculosis seen at the HHTMC, latent tuberculosis infection is largely treated by primary care providers in the Harris Health System and not referred to our clinic.

Evaluation of extrapulmonary tuberculosis cases in the United States from 1993 to 2006 demonstrated that 40.4% of all cases were lymphatic tuberculosis. ^20^ Similarly, in an analysis of extrapulmonary tuberculosis cases in Texas, lymphadenitis accounted for 32.3% of cases in patients without concomitant pulmonary tuberculosis and 20.8% among those with concomitant pulmonary tuberculosis, nearly identical to the prevalence of tuberculosis lymphadenitis observed in the HHTMC. ^21^

### Neurocysticercosis.

Although *Taenia solium* is rarely associated with autochthonous transmission in the United States as a result of pork processing regulations by the U.S. Department of Agriculture, ^22^ several thousand new cases of NCC are diagnosed in the United States annually. ^23^ Most of these cases were likely exposed inadvertently to *T. solium* eggs via fecal–oral transmission in endemic countries. There are at least three studies evaluating cases of NCC retrospectively at Ben Taub General Hospital, the largest hospital in the Harris Health System. In the most recent analysis of NCC in Houston published in 2011, 111 cases were diagnosed between 1997 and 2005, and 93% of these patients were foreign-born. ^7^ In our study, we describe 78 cases of NCC managed at the HHTMC between 2011 and 2020. This number likely underrepresents the actual disease burden in our region for several reasons, including there are no formal screening protocols for patients at risk of developing NCC, NCC is typically asymptomatic for decades, radiologists and clinicians unfamiliar with NCC may misinterpret characteristic imaging findings, and many cases of NCC are managed inpatient without outpatient referral.

### Strongyloidiasis.

A 2020 study conducted in a Central Texas community identified 16 seropositive individuals (16.5%) of 97 tested. ^24^ In immunocompromised patients at the University of Texas M.D. Anderson Cancer Center, one per 10,000 patients newly diagnosed with cancer between 1971 and 2003 had strongyloidiasis, ^25^ and an evaluation of at-risk solid organ transplant candidates observed a seroprevalence of nearly 10%. ^26^ Although most often diagnosed as asymptomatic intestinal disease, disseminated forms of strongyloidiasis can become fatal rapidly. ^27^ Thus, early diagnosis and treatment are critical to prevent significant parasite-induced morbidity and mortality, particularly in patients with impaired cellular immunity. In our study, we describe 28 cases of strongyloidiasis seen at the HHTMC. However, it is not uncommon for Harris Health primary care providers to manage asymptomatic *S. stercoralis* infection without subspecialty referral; thus, this number likely underrepresents the true disease burden in the Houston area.

### Chagas disease.

Although no national seroprevalence study of Chagas disease has been performed to date, the American Association of Blood Banks Chagas Biovigilance Network (which was established in 2007 to screen blood donors for *Trypanosoma cruzi*) reported 2,462 confirmed Chagas disease diagnoses between 2007 and 2019. ^15^ National estimates of Chagas disease prevalence vary widely, ranging from about 200,000 to nearly 1 million cases. ^28^^,^ ^29^ In Texas alone, some estimates suggest that as many as 250,000 people have Chagas disease. ^30^ Although most people with Chagas disease likely acquired the disease after exposure in an endemic country, triatomine “kissing bugs” (the insect vectors of *T. cruzi*) are endemic in the southern United States. Several reports have documented *T. cruzi*-positive vectors and mammalian reservoirs such as dogs and coyotes along the Texas–Mexico border regions. ^31^ It is increasingly apparent that autochthonous *T. cruzi* transmission occurs. Indeed, a study from 2015 reported five patients with natively acquired infections in Southeast Texas. ^32^ We describe 25 cases of Chagas disease seen at the HHTMC. Large seroprevalence studies and comparative studies of screening and diagnostic tests in at-risk populations are needed. Because *T. cruzi* infection can be transmitted vertically, studies of prenatal screening for *T. cruzi* to detect congenital Chagas disease are also needed. ^33^

### Schistosomiasis.

Data regarding schistosomiasis infection in the United States are extremely limited. Most existing studies examine the prevalence of schistosomiasis in at-risk groups such as refugees and people with HIV. Schistosomiasis is not endemic in the United States; therefore, cases detected in the United States are either acute disease in travelers or chronic infections in immigrants from endemic countries. More than 8,000 people are estimated to be living with schistosomiasis in the United States, mostly African refugees, based on seroprevalence testing among refugee populations. ^29^ In a study of Sudanese refugees living in the United States, 44% were seropositive for schistosomiasis. ^34^ Harris County is a major site for refugee resettlement: 25 of every 1,000 refugees worldwide resettle in this region. ^35^ Thus, schistosomiasis is likely present within our community but remains largely unrecognized. We describe only 12 cases of schistosomiasis seen at the HHTMC. This highlights the need for routine screening of all at-risk individuals to prevent morbidity from schistosomiasis-related conditions such as bladder cancer and pulmonary hypertension.

### Limitations.

Our study aimed simply to characterize the scope of patients presenting to the HHTMC. Even so, this study had several limitations. Because we identified patients using the schedules of only three physicians who regularly saw patients during the study time period, the patients do not represent a comprehensive cohort of the HHTMC patients seen during this time period. In addition, referral bias and selection bias were introduced because of this patient identification method. Because of its retrospective nature, data collection was dependent on the quality of information provided by the clinicians. For example, certain elements of the exposure history were collected and documented for some patients with a given diagnosis but not others. Travel histories and time spent living in the United States were not documented consistently by providers and were therefore unknown for many patients. In addition, although we recorded time in the United States for patients seen in the clinic, this measure alone does not consider separate risk factors such as travel history or visitors from abroad. Many patients had lived in Houston for years or decades, making it impossible to determine whether their infections were acquired locally or internationally when the causative pathogen is endemic in Texas as well as in their country of origin/travel. Another limitation was the degree of detail we were able to reach for certain data points. For example, we were unable to distinguish between patients with Medicare or Medicaid and patients with private employment-based health insurance because of the format of the electronic medical record. The HHTMC is a relatively new clinic model designed to support health-care providers with the diagnosis and management of tropical diseases in the Houston region. However, other providers in the region do diagnose and treat tropical diseases without referral to the HHTMC; thus, the data presented here represents a small and biased proportion of the true burden of tropical diseases found in the Houston area. This further supports the ongoing need for the HHTMC and increased awareness of the services it provides.

## CONCLUSION

Although tropical diseases have received increasing international attention since 2005, when policymakers formally recognized the global impact of NTDs, ^36^ large-scale study of tropical diseases in the United States remains limited. ^37^ By detailing the frequencies of tropical disease diagnoses at the HHTMC, we begin the process of uncovering the true burden of tropical disease prevalence in our region. The data presented here underscore high-yield public health targets for tropical disease epidemiological research in the United States. Furthermore, this study demonstrates the importance of establishing outpatient tropical medicine clinics throughout the United States, especially in areas of relatively high disease prevalence such as the Gulf Coast, Appalachia, and urban areas that are not only endemic for tropical diseases, but are also home to large populations of immigrants and refugees. Although patients with tropical diseases can receive appropriate initial treatment in hospitals with the help of infectious diseases specialists, many tropical diseases are chronic and require long-term outpatient follow-up with knowledgeable subspecialists. Such clinics must be inexpensive and easily accessible to both recent immigrants and long-term residents of the United States. In addition, development of comprehensive, systematic national tropical disease screening programs and patient registries will be key to improving tropical disease care in the United States. Such studies will expand our understanding of the natural history of tropical diseases and the nuances of their management in the United States. In parallel with large-scale screening and seroprevalence studies, active analysis of individual and cumulative tropical disease cases at clinics that care for patients with tropical diseases, as we have done here, will advance efforts to improve our understanding of the epidemiology and risk factors for tropical diseases in the United States.

Last, a major obstacle to tropical disease recognition and management in the United States is front-line health-care providers’ limited tropical disease knowledge base. Tropical disease curricula should be expanded in health-care provider training programs to aid in disease recognition, particularly in parts of the country where tropical diseases are common. Such a provider training curriculum is available through Baylor College of Medicine’s NSTM. It trains learners from diverse backgrounds and has accessible basic, translational, and clinical research platforms to provide instruction on research development in tropical medicine alongside clinical training. Training front-line health-care providers to recognize and manage tropical diseases—including timely referral to subspecialists—will greatly improve outcomes for tropical disease patients in the United States.

## Supplemental Material


Supplemental materials

## References

[1] Centers for Disease Control and Prevention, Division of Parasitic Diseases and Malaria , 2021. *Neglected Tropical Diseases*. Available at: https://www.cdc.gov/globalhealth/ntd/. Accessed January 31, 2022.

[2] Kyu HH Abate D Abate KH Abay SM Abbafati C Abbasi N Abbastabar H Abd-Allah F Abdela J Abdelalim A , 2018. Global, regional, and national disability-adjusted life-years (DALYs) for 359 diseases and injuries and healthy life expectancy (HALE) for 195 countries and territories, 1990–2017: a systematic analysis for the Global Burden of Disease Study 2017. Lancet 392: 1859–1922. 30415748 10.1016/S0140-6736(18)32335-3 PMC6252083

[3] Hotez P , 2013. *The Disease Next Door*. Available at: https://foreignpolicy.com/2013/03/25/the-disease-next-door/. Accessed January 31, 2022.

[4] Hotez PJ , 2013. NTDs V. 2.0: “Blue Marble Health”: neglected tropical disease control and elimination in a shifting health policy landscape. PLoS Negl Trop Dis 7: e2570. 24278496 10.1371/journal.pntd.0002570 PMC3836998

[5] Hotez PJ , 2018. The rise of neglected tropical diseases in the “new Texas”. PLoS Negl Trop Dis 12: e0005581. 29346369 10.1371/journal.pntd.0005581 PMC5773009

[6] Nolan MS Aguilar D Brown EL Gunter SM Ronca SE Hanis CL Murray KO , 2018. Continuing evidence of Chagas disease along the Texas–Mexico border. PLoS Negl Trop Dis 12: e0006899. 30427833 10.1371/journal.pntd.0006899 PMC6261633

[7] Serpa JA Graviss EA Kass JS White AC Jr , 2011. Neurocysticercosis in Houston, Texas: an update. Medicine (Baltimore) 90: 81–86. 21200189 10.1097/MD.0b013e318206d13e

[8] Henderson H , 1957. Incidence and intensity of hookworm infestation in certain east Texas counties with comparison of technics. Tex Rep Biol Med 15: 283–291. 13433631

[9] Centers for Disease Control and Prevention , 2020. *Tuberculosis.* Available at: www.cdc.gov/tb/statistics/default.htm. Accessed January 31, 2022.

[10] Rouphael NG Talati NJ Vaughan C Cunningham K Moreira R Gould C , 2007. Infections associated with haemophagocytic syndrome. Lancet Infect Dis 7: 814–822. 18045564 10.1016/S1473-3099(07)70290-6 PMC7185531

[11] Centers for Disease Control and Prevention , 2020. *Parasites: Strongyloides*. Available at: www.cdc.gov/parasites/strongyloides/gen_info/faqs.html. Accessed January 31, 2022.

[12] Starr MC Montgomery SP , 2011. Soil-transmitted helminthiasis in the United States: a systematic review: 1940–2010. Am J Trop Med Hyg 85: 680–684. 21976572 10.4269/ajtmh.2011.11-0214 PMC3183777

[13] Vohra RF Walker DH Blanton LS , 2018. Analysis of health-care charges in murine typhus: need for improved clinical recognition and diagnostics for acute disease. Am J Trop Med Hyg 98: 1594–1598. 29637877 10.4269/ajtmh.17-0411 PMC6086180

[14] Capps R Ruiz Soto AG , 2018. A Profile of Houston’s Diverse Immigrant Population in a Rapidly Changing Policy Landscape. Washington, DC: Migration Policy Institute.

[15] Hotez P , 2014. Tropical medicine in the horse latitudes. Curr Trop Med Rep 1: 3–5. 32226713 10.1007/s40475-013-0003-6 PMC7100063

[16] Harris Health System , 2021. *Patient Eligibility*. Available at: https://www.harrishealth.org/access-care/patient-eligibility. Accessed March 10, 2021.

[17] Centeno FH Lasco T Ahmed AA Al Mohajer M , 2021. Characteristics of *Rickettsia typhi* infections detected with next-generation sequencing of microbial cell-free DNA in a tertiary care hospital. *Open Forum Infect Dis* *8:* ofab147. 10.1093/ofid/ofab147 PMC8266567 34250186

[18] Mancuso JD Diffenderfer JM Ghassemieh BJ Horne DJ Kao T-C , 2016. The prevalence of latent tuberculosis infection in the United States. Am J Respir Crit Care Med 194: 501–509. 26866439 10.1164/rccm.201508-1683OC PMC12057332

[19] Bibbins-Domingo K Grossman DC Curry SJ Bauman L Davidson KW Epling JW García FA Herzstein J Kemper AR Krist AH , 2016. Screening for latent tuberculosis infection in adults: US Preventive Services Task Force recommendation statement. JAMA 316: 962–969. 27599331 10.1001/jama.2016.11046

[20] Peto HM Pratt RH Harrington TA LoBue PA Armstrong LR , 2009. Epidemiology of extrapulmonary tuberculosis in the United States, 1993–2006. Clin Infect Dis 49: 1350–1357. 19793000 10.1086/605559

[21] Qian X Nguyen DT Lyu J Albers AE Bi X Graviss EA , 2018. Risk factors for extrapulmonary dissemination of tuberculosis and associated mortality during treatment for extrapulmonary tuberculosis. Emerg Microbes Infect 7: 1–14. 29872046 10.1038/s41426-018-0106-1 PMC5988830

[22] Centers for Disease Control and Prevention , 2020. *Parasites: Taeniasis*. Available at: www.cdc.gov/parasites/taeniasis/gen_info/faqs.html. Accessed January 31, 2022.

[23] Serpa JA Clinton White A , 2012. Neurocysticercosis in the United States. Pathog Glob Health 106: 256–260. 23265549 10.1179/2047773212Y.0000000028 PMC4005108

[24] Singer R Xu TH Herrera LNS Villar MJ Faust KM Hotez PJ Aiken AR Mejia R , 2020. Prevalence of intestinal parasites in a low-income Texas community. Am J Trop Med Hyg 102: 1386–1395. 32207401 10.4269/ajtmh.19-0915 PMC7253135

[25] Safdar A Malathum K Rodriguez SJ Husni R Rolston KV , 2004. Strongyloidiasis in patients at a comprehensive cancer center in the United States. Cancer 100: 1531–1536. 15042689 10.1002/cncr.20120

[26] Clark E Pritchard H Hemmige V Restrepo A Bautista K Damania A Ricciardi A Nutman TB Mejia R , 2020. *Strongyloides stercoralis* infection in solid organ transplant patients is associated with eosinophil activation and intestinal inflammation: a cross-sectional study. Clin Infect Dis 71: e580–e586. 32155244 10.1093/cid/ciaa233 PMC7744999

[27] Croker C Reporter R Redelings M Mascola L , 2010. Strongyloidiasis-related deaths in the United States, 1991–2006. Am J Trop Med Hyg 83: 422–426. 20682893 10.4269/ajtmh.2010.09-0750 PMC2911196

[28] Bern C Kjos S Yabsley MJ Montgomery SP , 2011. *Trypanosoma cruzi* and Chagas’ disease in the United States. Clin Microbiol Rev 24: 655–681. 21976603 10.1128/CMR.00005-11 PMC3194829

[29] Hotez PJ , 2008. Neglected infections of poverty in the United States of America. PLoS Negl Trop Dis 2: e256. 18575621 10.1371/journal.pntd.0000256 PMC2430531

[30] Hanford EJ Zhan FB Lu Y Giordano A , 2007. Chagas disease in Texas: recognizing the significance and implications of evidence in the literature. Soc Sci Med 65: 60–79. 17434248 10.1016/j.socscimed.2007.02.041

[31] Garcia MN O’Day S Fisher-Hoch S Gorchakov R Patino R Feria Arroyo TP Laing ST Lopez JE Ingber A Jones KM , 2016. One health interactions of Chagas disease vectors, canid hosts, and human residents along the Texas–Mexico border. PLoS Negl Trop Dis 10: e0005074. 27832063 10.1371/journal.pntd.0005074 PMC5104435

[32] Garcia MN Aguilar D Gorchakov R Rossmann SN Montgomery SP Rivera H Woc-Colburn L Hotez PJ Murray KO , 2014. Case report: evidence of autochthonous Chagas disease in southeastern Texas. *Am J Trop Med Hyg 92: *325--330. 10.4269/ajtmh.14-0238 PMC4347336 25371187

[33] Stillwaggon E Perez-Zetune V Bialek SR Montgomery SP , 2018. Congenital Chagas disease in the United States: cost savings through maternal screening. Am J Trop Med Hyg 98: 1733–1742. 29714163 10.4269/ajtmh.17-0818 PMC6086189

[34] Posey DL Blackburn BG Weinberg M Flagg EW Ortega L Wilson M Secor WE Sanders-Lewis K Won K Maguire JH , 2007. High prevalence and presumptive treatment of schistosomiasis and strongyloidiasis among African refugees. Clin Infect Dis 45: 1310–1315. 17968826 10.1086/522529

[35] Kragie A , 2017. *City of Refugees: How Houston Became a Resettlement Magnet*. Available at: https://www.houstonchronicle.com/local/gray-matters/amp/The-refugee-who-welcomes-the-refugees-6468958.php. Accessed March 10, 2021.

[36] Hotez PJ Aksoy S Brindley PJ Kamhawi S , 2020. World Neglected Tropical Diseases Day. San Francisco, CA: PLoS Negl Trop Dis. 10.1371/journal.pntd.0007999 PMC6988912 31995572

[37] Hotez PJ , 2017. The poverty-related neglected diseases: why basic research matters. PLoS Biol 15: e2004186. 29121043 10.1371/journal.pbio.2004186 PMC5679514

