# Supplementary material for: Reviewing a Decade of Outpatient Tropical Medicine in Houston, Texas
Source: Am J Trop Med Hyg. 2022 Feb 28;106(4):1049–56. doi: 10.4269/ajtmh.21-1059 (PMC8991353; doi:10.4269/ajtmh.21-1059)

Supplemental Table 1. Details regarding top five diagnoses seen overall.

Disease	Age (median, range)	Ethnicity	Continent of origin	Insurance status
**Tuberculosis, extrapulmonary and latent (n=169)**	47, 19-83	88 (52.1%) Hispanic<br>41 (24.3%) Black<br>13 (7.7%) Asian<br>12 (7.1%) Middle Eastern<br>4 (2.4%) White<br>6 (3.6%) Other<br>5 (3.0%) Unknown	86 (50.9%) Latin America<br>31 (18.3%) Asia<br>23 (13.6%) North America<br>22 (13.0%) Africa<br>1 (5.9%) Europe<br>6 (3.6%) Unknown	90 (53.3%) Gold Card<br>66 (3.9%) Private<br>28 (16.6%) Self-pay<br>16 (9.5%) Other
**Neurocysticercosis (n=78)**	39.5, 21-89	77 (98.7%) Hispanic<br>1 (1.3%) Other	75 (96.2%) Latin America<br>1 (1.3%) North America<br>2 (2.6%) Unknown	35 (44.9%) Gold Card<br>24 (30.7%) Private<br>17 (21.8%) Self-pay<br>5 (6.4%) Other
**Strongyloidiasis (n=28)**	49, 25-76	24 (85.7%) Hispanic<br>2 (7.1%) Black	24 (85.7%) Latin America	12 (42.8%) Gold Card

		1 (3.6%) Asian<br>1 (3.6%) White	2 (7.1%) Africa<br>1 (3.6%) Asia<br>1 (3.6%) North America	10 (35.7%) Private<br>7 (25.0%) Self-pay<br>1 (3.6%) Other
**Chagas disease (n=25)**	45, 20-74	22 (88.0%) Hispanic<br>3 (12.0%) White	22 (88.0%) Latin America<br>2 (8.0%) North America<br>1 (4.0%) Unknown	10 (40.0%) Private<br>9 (36.0%) Self-pay<br>8 (32.0%) Gold Card
**Schistosomiasis (n=12)**	39.5, 24-75	9 (75.0%) African American<br>1 (8.3%) Middle Eastern<br>1 (8.3%) White<br>1 (8.3%) Asian	10 (83.3%) Africa<br>1 (8.3%) Asia<br>1 (8.3%) North America	7 (58.3%) Gold Card<br>3 (25.0%) Private<br>3 (25.0%) Self-pay

Supplemental Fig 1: Patients seen per year at the Tropical Medicine Clinic.

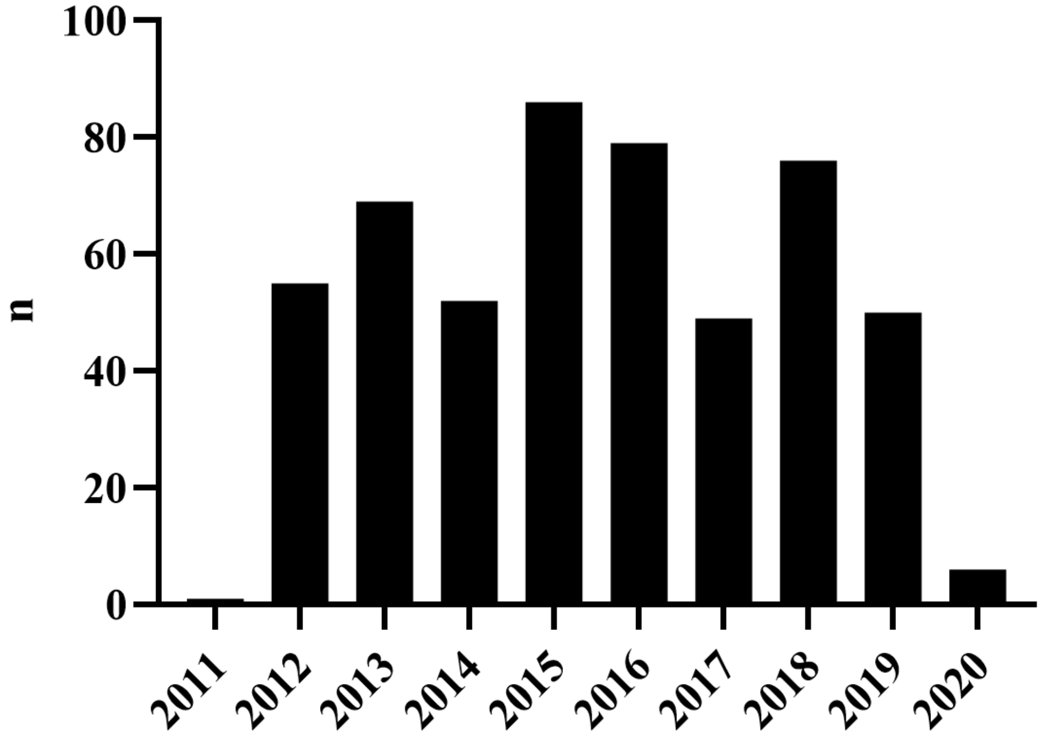

Supplement: Supplementary file 1 [file tpmd211059.SD1.pdf]
